# Acetaminophen enhances the reflective learning process

**DOI:** 10.1093/scan/nsy074

**Published:** 2018-08-24

**Authors:** Rahel Pearson, Seth Koslov, Bethany Hamilton, Jason Shumake, Charles S Carver, Christopher G Beevers

**Affiliations:** 1 Department of Psychology and Institute for Mental Health Research, University of Texas at Austin, Austin, USA; 2 Department of Psychology, University of Miami, Coral Gables FL

**Keywords:** acetaminophen, learning, categorization, COVIS, psychopharmacology

## Abstract

Acetaminophen has been shown to influence cognitive and affective behavior possibly via alterations in serotonin function. This study builds upon this previous work by examining the relationship between acetaminophen and dual-learning systems, comprising reflective (rule-based) and reflexive (information-integration) processing. In a double-blind, placebo-controlled study, a sample of community-recruited adults (*N* = 87) were randomly administered acetaminophen (1000 mg) or placebo and then completed reflective-optimal and reflexive-optimal category learning tasks. For the reflective-optimal category learning task, acetaminophen compared to placebo was associated with enhanced accuracy prior to the first rule switch (but not overall accuracy), with needing fewer trials to reach criterion and with a faster learning rate. Acetaminophen modestly attenuated performance on the reflexive-optimal category learning task compared to placebo. These findings indirectly support two positions that have been proposed elsewhere. First, they are consistent with the view that acetaminophen has an influence on the serotonergic system. Second, the findings are consistent with a proposed link between elevated serotonin function and relative dominance of effortful, rule-based processing.

## Introduction

Acetaminophen is the active ingredient in many over-the-counter pain relief medications, which are taken by more than 50 million people weekly (Kaufman *et al.*, 2002). Despite widespread use, there is still uncertainty about acetaminophen’s mechanisms of action (see Graham *et al.*, 2013 for a review).

One of the leading hypotheses suggests that the analgesic effect of acetaminophen can be attributed to the promotion of the activity of the descending inhibitory serotonin pathway (Pickering *et al.*, 2008). Various spinal serotonin receptors have been implicated in acetaminophen’s mechanism of action, including Serotonin 1B, and Serotonin 2A and 2C receptors (Courade *et al.*, 2001). A down regulation of serotonin receptors has been observed in the brains of rodents who were administered acetaminophen, suggesting increases in central serotonin (Pini *et al.*, 1996; Srikiatkhachorn *et al.*, 2000). This aligns with other evidence of increased availability of serotonin following acetaminophen administration (Pini *et al*., 1996; Courade *et al*., 2001). Perhaps as a consequence of these increases in central serotonin, acetaminophen intake has also been shown to increase the number of serotonin transporter binding sites, suggesting enhanced serotonin reuptake (Srikiatkhachorn *et al*., 2000).

These findings raise the intriguing possibility that acetamino-phen intake may affect other serotonin-mediated psychological processes. Low serotonergic function has been linked to aggressive and violent behavior for some time (see Carver and Miller, 2006 for review). But there is evidence that the association depends heavily on habitual tendencies rather than on aggression *per se*. That is, Cleare and Bond (1995) assessed participants as being either high or low in aggressive tendencies. Those high in aggressive tendencies became more aggressive, hostile, and quarrelsome after tryptophan depletion and less so after tryptophan enhancement. Tryptophan manipulation had no effect on behavior for those low in aggressive tendencies. A later study (Bjork *et al.*, 2000) further reinforced this point: tryptophan depletion led to greater aggressive responses to provocation among men high in aggressiveness but had an opposite effect among those low in aggressiveness. Similar results were reported by Finn and colleagues (Finn *et al.*, 1998).

This pattern suggests that effects of low serotonergic function on aggression are less about aggression *per se* and more about the reliance on existing habitual tendencies to be aggressive (see also Spoont, 1992; Manuck *et al.*, 2006). Conversely, enhanced serotonergic function might promote effortful control over underlying action tendencies (as argued by Spoont, 1992; Carver *et al.*, 2008).

Several studies involving acetaminophen, which putatively enhances serotonergic function, provide evidence consistent with this possibility. For example, persons who receive acetaminophen display blunted evaluative responses to both positive and negative stimuli (Durso *et al.*, 2015), similar to what is seen with higher serotonergic function (Spoont, 1992; Depue, 1995). Acetaminophen has also been shown to decrease sensitivity to social rejection (DeWall *et al*., 2010), scenarios that evoke distrust (Roberts *et al.*, 2017) and pain that others experience (Mischkowski *et al.*, 2016).

Taken together, findings involving responses to emotion-related contexts are consistent with the idea that enhancing serotonin function via acetaminophen should decrease reliance on habitual, reflexive forms of self-regulation and increase the use of effortful, reflective forms of behavior regulation. This would be in line with theoretical accounts that emphasize the contribution of serotonergic function to these two modes of behavioral regulation (for reviews, see Carver *et al*., 2008, 2009). A direct implication of this work, then, is that acetaminophen (via enhanced serotonergic function) may affect other domains of behavior regulation in which dual processes have been implicated, such as category learning. This is an ideal candidate for studying the effects of acetaminophen, as there is a good deal of support for dual-system models of category learning and a role for serotonin in differentiating these systems (Smith and DeCoster, 2000; Ashby and Maddox, 2005).

One framework for describing the underlying mechanisms supporting learning and decision-making is the COmpetition between Verbal and Implicit Systems model (COVIS; Ashby and Alfonso-Reese, 1998; Ashby *et al.*, 2011). COVIS is a biologically constrained model of learning and decision-making that posits two dissociable systems—reflective (also referred to as rule-based) and reflexive (also referred to as information-integration)—operate simultaneously to generate a response based on environmental inputs. Output control for observable behaviors is modulated between the two systems based on continuing competition between the systems for influence (Paul and Ashby, 2013). Due to the competition between systems, enhanced performance in one can impair performance in the other (Ashby and Maddox, 2011). Relative dominance of the reflective system *vs* the reflexive system can be tested experimentally by use of categorization-learning tasks where optimal performance requires engagement of one of the two processing systems.

Categorization on reflective-optimal tasks depends on logical reasoning processes (e.g. hypothesis testing). Rules on these tasks are easily verbalized and individuals maximize accuracy by using an explicit hypothesis and iterative hypothesis updating to guide categorization. For example, rule-based categorization tasks like the oft-used Wisconsin Card Sorting Task (WCST) are thought to rely on reflective learning processes (Hélie *et al.*, 2012). In contrast, categorization learning on reflexive-optimal tasks depends on intuitive processing and cumulative, incremental reinforcement learning. Rules on these tasks are not easily verbalized. Accuracy on reflexive-optimal tasks is maximized by integrating high-level perceptual details (e.g. height and color) at a pre-decisional stage and associating those percepts with rewarding motor actions.

The involvement of the serotonergic system in the balance between these competing systems has been supported by a study of the relationship between the serotonin transporter gene-linked promoter region (5-HTTLPR)and category learning (Maddox *et al*., 2017). Individuals with the low-expressing variants of 5-HTTLPR (S/L_G_) have significantly lower serotonin transporter function, binding and messenger RNA (mRNA) expression than individuals homozygous for the high-expressing variant (L_A_), leading to decreases in the reuptake of serotonin and increases in extracellular serotonin availability (Collier *et al*., 1996). As predicted by the reasoning outlined above, persons with low-expressing variants of 5-HTTLPR (S/L_G_) had enhanced performance (compared to high-expressing L_A_ homozygotes) on tasks relying on the reflexive systems, whereas L_A_ homozygotes (compared to S/L_G_ carriers) had enhanced performance on tasks relying on the reflective system (Maddox *et al*., 2017).

The studies in Maddox *et al*. (2017) provide preliminary evidence for an association between serotonergic neurotransmission and dual-learning systems. However, these results need to be interpreted with caution since candidate gene studies are especially vulnerable to spurious associations caused by unmeasured third variables (Tabor *et al.*, 2002; Thomas and Witte, 2002; Dick, 2005). Rather than relying on genetically mediated individual differences in serotonergic function, the current study experimentally manipulated acetaminophen intake that, as reviewed above, has been shown to influence serotonergic availability and function (Pini *et al*., 1996; Srikiatkhachorn *et al*., 2000; Courade *et al*., 2001).

Consistent with the idea that enhanced serotonin function promotes control over habitual action tendencies (Carver *et al*., 2009), we anticipated that acetaminophen would enhance effortful, reflective learning and decrease reliance on intuitive, reflexive learning strategies. Thus, category learning performance following acetaminophen would be similar to the performance of L_A_ carriers of 5-HTTLPR.

## Methods

### Participants

One hundred community participants were recruited through online ads, university listservs and flyers, and were compensated $10 per hour for study participation. Included participants were between the ages of 18 and 35 and did not endorse significant depressive symptoms (score ≤ 15 on Center for Epidemiologic Studies Depression Scale; Radloff, 1977) or contraindications for acetaminophen intake. Six participants (four active and two placebo) were excluded from analyses because they lacked complete task data, and seven participants (five active and two placebo) were excluded from analyses because their category-task performance was at or below chance (≤50%). The remaining 87 participants (age mean = 20.37, s.d.= 2.48, 59% female) were 54% Caucasian, 31% Asian, 8% African American and 7% mixed race. Demographic variables did not significantly differ between the active and placebo group.

### Experimental manipulation

Participants were instructed to refrain from using alcohol and medications containing acetaminophen the night prior to and the day of the experiment. To aid drug absorption, participants were asked to abstain from eating 3 h prior to the experiment. Participants were randomized to receive a 1000 mg of acetaminophen dissolved in flavored suspension liquid or a placebo consisting of identical looking and tasting flavored suspension liquid. The placebo and acetaminophen suspensions were prepared by a pharmacist at the Austin Compounding Pharmacy in Austin, Texas. Participants and the experimenters were blind to drug condition. Participants were randomly assigned to drug condition by the experimenter using a random number generator. The experimenter was unaware of which drug condition participants were assigned, as the suspensions were labeled by the pharmacist as suspension A and suspension B.

### Category learning tasks

For the reflective-optimal and reflexive-optimal tasks, participants were presented with a single stimulus and asked to indicate whether it belonged in category A or B by pressing a corresponding response button. Participants received feedback after each response that indicated whether their response was correct or incorrect.

**Fig. 1:**
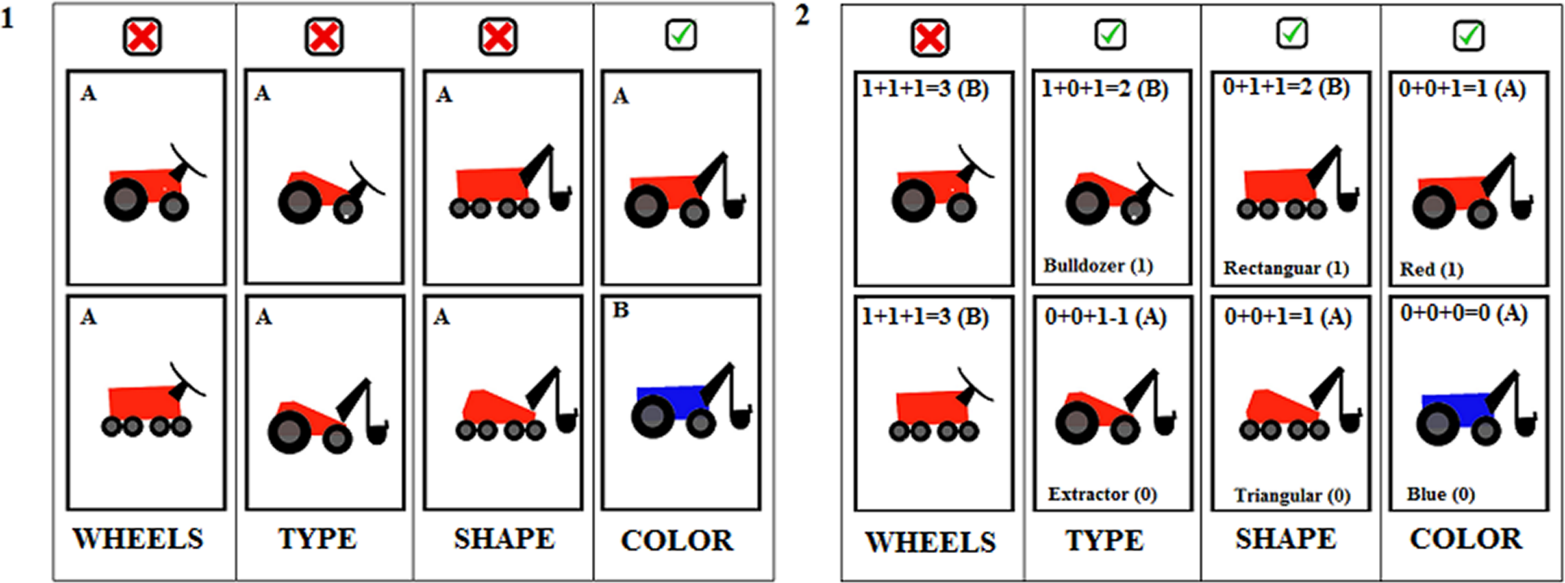
**(1)** An example of the reflective task where the dimension color is used to separate categories, and the other dimensions (wheels, type and shape) are irrelevant. **(2)** An example of a reflexive task where the dimensions type, shape and color are used to separate categories, and the wheels dimension is irrelevant.

Two sets of stimuli containing images of houses or vehicles were used. Images in each set differed along four dimensions: vehicles varied in shape (triangle *vs* square), number of visible wheels (two *vs* four), type (bulldozer *vs* excavator) and color (red *vs* blue); whereas houses varied in window shape (square *vs* round), number of clouds (one *vs* two), accessory (tree *vs* bush) and color (green *vs* pink). Each set contained 16 unique images of vehicles or houses generated from the factorial combination of possible features.

For the reflective-optimal task, one dimension was chosen to separate categories (e.g. house color: green = category A, pink = category B) and after 10 consecutive correct categorizations, the dimension that separates the categories changed (e.g. window shape: square = A, round = B). This categorization rule is easily verbalized, consistent with a reflective-optimal task. This task is a variant of the commonly used WCST.

For the reflexive-optimal task, one dimension (e.g. color) was made irrelevant. The properties of the remaining three dimensions were assigned a value of 0 or 1 (e.g. window shape: square = 0, round = 1) and summed. Category A was defined as a sum value of 0 or 1 and category B was defined as a sum property value of 2 or 3 (see Figure 1 for an example). This rule is not easily verbalized and instead depends on trial and error and reinforcement learning (see Maddox *et al*., 2017).

Participants completed 150 trials for each of the tasks. Participants were instructed that presented stimuli belonged to one of two categories (A or B) and that their task was to determine the correct category for each stimulus by pressing the ‘s’ key (category A) or the ‘l’ key (category B) on the computer keyboard. Stimuli remained on screen until a response was generated. Following the participant’s response, feedback stating whether the category was correct or incorrect was given for 1 s, followed by a 500 ms inter trial interval (ITI). Participants received no information about category properties and were instructed to use task feedback to improve their performance. Tasks were coded in Psychophysics Toolbox for MATLAB (Brainard and Vision, 1997; Pelli, 1997).

### Procedure

All procedures were completed at the University of Texas. Upon arrival in the laboratory, participants provided informed consent and consumed their randomly assigned compound. Consistent with previous research (Randles *et al.*, 2016), participants waited 60 min after ingesting the solution to allow for drug absorption. Participants then completed both the reflective-optimal and reflexive-optimal learning tasks in individual testing rooms using Dell computers with 1280 × 1024 resolution. Task order (reflective-optimal, reflexive-optimal) and stimuli set (vehicles, houses) were counterbalanced across participants. This study was approved by the Institutional Review Board at the University of Texas at Austin.

## Results

### Overall accuracy

A 2 × 2 mixed model analysis of variance (ANOVA) with experimental group (acetaminophen *vs* placebo) as a between-subject factor and task condition (reflective *vs* reflexive) as a within-subject factor was used to examine overall accuracy. We predicted a significant interaction between experimental group and task condition, where those in the acetaminophen group would have enhanced performance (*vs* placebo) in the reflective condition and poorer performance (*vs* placebo) in the reflective condition.

The interaction between task, condition and experimental group was not significant, *F*(1, 85) = 3.38, *P* = 0.07, η ^2^ = 0.02. Follow-up Welch test pairwise comparisons of the effect of drug condition on overall accuracy within the reflective [M = 0.65, s.d. = 0.08 in acetaminophen group; M = 0.62, s.d = 0.07 in placebo group; *t*(77.2) = −1.24, *P =* 0.22, *d* = 0.27] and reflexive [M = 0.61, s.d. = 0.08 in acetaminophen group; M = 0.64, s.d = 0.09 in placebo group; *t*(84.6) = 1.39, *P* = 0.17, *d* = 0.29] conditions were also not significant.

Recall that in the reflective-optimal condition, rule changes occurred after 10 consecutive correct responses. The timing of when the rule changes occurred were not uniform across participants, which means that some participants had to learn more rule changes than others. This could suppress overall task accuracy. Therefore, an exploratory analysis was conducted to examine accuracy until the first rule change in the reflective condition. Accuracy scores were examined in a similar 2 × 2 mixed model ANOVA, with experimental group (acetaminophen *vs* placebo) as a between-subject factor and task condition (reflective *vs* reflexive) as a within-subject factor. The interaction between task condition and experimental group was significant in this model, *F*(1, 85) = 5.41, *P* = 0.02, η ^2^ = 0.03. The Welch test indicated that the acetaminophen group (*vs* placebo) had greater accuracy before rule change [acetaminophen: M = 0.88, s.d. = 0.10; placebo: M = 0.82, s.d. = 0.17; *t*(74.7) = −2.00, *P =* 0.049, *d* = 0.42] in the reflective condition.

### Trials to criterion

Trials to criterion were operationalized as the number of trials needed to obtain 10 consecutive correct responses and were examined using a 2 × 2 mixed model ANOVA with experimental group as a between-subject factor and task condition as a within-subject factor. However, only a subset (N = 35) of participants reached criterion in the reflexive-optimal condition, whereas all participants reached criterion in the reflective-optimal condition. To complete the analyses, those who did not reach criterion were assigned the maximum number of trials (150).

We predicted a significant interaction between experimental group and task condition on trial to criterion, where those in the acetaminophen group (*vs* placebo) would reach criterion faster in the reflective-optimal condition and those in the placebo group (*vs* acetaminophen) would reach criterion faster in the reflexive-optimal condition. The interaction between task condition and experimental group was not significant, *F*(1, 85) = 3.64, *P* = 0.06, η ^2^ = 0.02. Follow-up Welch test pairwise comparisons indicated that participants in the acetaminophen group (M = 14.8, s.d. = 4.4) reached criterion faster than participants in the placebo group (M = 18.8, s.d. = 9.4) in the reflective-optimal task condition, *t*(65.4) = −2.64, *P =* 0.01, *d* = 0.54. In the reflexive condition, there was no significant difference in trials to criterion between experimental groups [M = 132.4, s.d. = 35.7 in acetaminophen group; M = 119.8, s.d. = 40.9 in placebo group; *t*(84.9) = 1.53, *P =* 0.13, *d* = 0.32].

Since the majority of participants failed to reach criterion in the reflexive-optimal condition, an exploratory analysis was conducted to examine the influence of acetaminophen on reaching criterion (yes *vs* no) in the reflexive condition. For the reflexive-optimal task, those receiving placebo (*n* = 23) had a higher likelihood of reaching criterion than those receiving acetaminophen (*n* = 12), which was significant x^2^(1, *N* = 87) = 3.9, *P* = 0.049.

### Learning rate

**Fig. 2:**
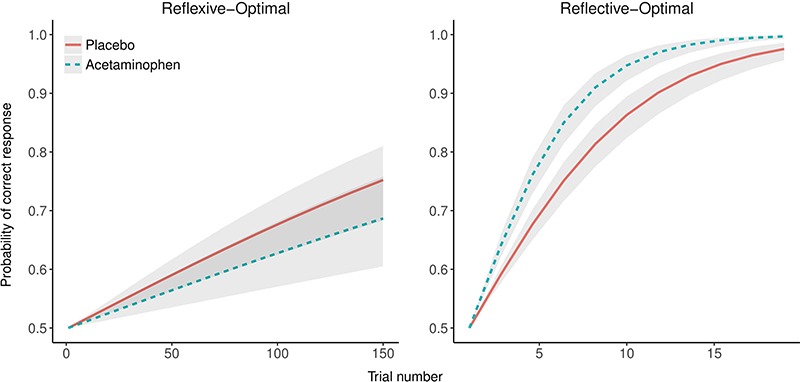
Relationship between learning rate and task condition presented as function of acetaminophen exposure.

Learning rate is the change in probability of generating a correct response as a function of trial number. Because initial response accuracy on the first trial must necessarily be due to chance, no participant should be predicted to have a higher or lower accuracy rate than 50% on the first trial. Thus, we assume a fixed intercept (at 50% probability of correct response) with a random slope of trial given participant and task. Fixed effects were specified for trial number, experimental group and task condition. This model supported a significant three-way interaction between trial number, experimental group and task condition, b = 2.39, SE = 0.57, z = 4.22, *P* < 0.0001^1^; odds ratio at maximum observed group difference (reflective) = 1.1; odds ratio at maximum observed group difference (reflexive) = 0.91. As can be seen in Figure 2, there was a non-significant difference between the drug conditions in the reflexive-optimal condition; the placebo condition tended to learn faster than the acetaminophen condition. However, in the reflective-optimal condition, the acetaminophen condition had a significantly faster learning rate to criterion than the placebo condition, as the 95% confidence intervals (CIs) were non-overlapping for the majority of the trials.

## Discussion

This study examined whether acetaminophen altered participants’ performance on reflective-optimal and reflexive-optimal category learning tasks. Findings indicated that acetaminophen (*vs* placebo) enhanced performance on a category learning task in which reflective learning was optimal. Specifically, on this task, acetaminophen was associated with enhanced accuracy prior to the first rule switch (though not overall accuracy), with needing fewer trials to reach criterion and with a faster learning rate. For the reflexive-optimal task, acetaminophen tended to impair performance, as participants in the placebo condition were more likely to reach the learning criterion and their learning rate was modestly faster than those in the acetaminophen condition; however, the effects of acetaminophen were less pronounced in the reflexive-optimal than reflective-optimal task condition.

Findings from this study have two important, albeit indirect, conceptual implications. The first concerns the potential role of serotonergic function in acetaminophen effects. The influence of acetaminophen in the study reported here aligns with prior work examining genetic variation in the 5-HTTLPR and dual-system category learning (Maddox *et al*., 2017). That work showed that 5-HTTLPR L_A_ homozygotes, who putatively have greater serotonin transport functionality, outperformed S/L_G_ allele homozygotes in a reflective-optimal learning task and performed more poorly than the comparison group on the reflexive-optimal learning task. The fact that the same pattern emerged here for acetaminophen indicates the plausibility of involvement of a serotonergic pathway in diverse acetaminophen effects.

The second implication of the pattern of results concerns the link between the tasks and the conceptual framework of dual-process models. It is widely argued that there are two competitive modes of learning, one model-based (reflective), the other model-free (reflexive). To the extent that predictor variables differentiate between the two forms of learning, results from such studies tend to support the plausibility of the dual-process framework. In the study reported here, acetaminophen enhanced reflective-optimal learning (and to a lesser degree impaired reflexive-optimal learning). Notably, there have been proposals that differences in serotonergic function lead to effects that are readily interpreted in terms of dual-process models (Carver *et al*., 2008, 2009). The present results are consistent with that view.

To the degree that people who take acetaminophen are trying to make decisions with clear, logical rules, their performance may improve. For decisions that require more implicit forms of learning, their performance may suffer. Indeed, these findings are consistent with other research which indicates that acetaminophen may attenuate emotional reactivity to personal and other people’s pain (Mischkowski *et al*., 2016) and possibly blunt emotional responses to affective stimuli in general (Durso *et al*., 2015). Although speculative, acetaminophen could potentially help people make difficult decisions by reducing emotional responses to affective contexts while at the same time facilitating more deliberative, effortful information processing (DeWall *et al.*, 2010).

There are certainly limitations on these inferences. Other neurotransmitter systems are thought to be involved in effects of acetaminophen, and changes have been reported in eicosanoid, opioid, cannabinoid, cyclooxygenase and nitric oxide systems (Anderson, 2008; Graham *et al.*, 2013). Further, it seems likely that these systems interact in ways not yet fully defined. Therefore, these results can potentially be fully or partially attributed to mechanisms other than serotonin. Research using additional pharmacological approaches (e.g. tryptophan manipulations) may be especially useful to provide further convergent evidence on the contribution of serotonergic function to dual-system learning. For instance, acute tryptophan depletion in humans has been shown to shift behavior toward habitual, reinforcement-based learning and away from goal-directed, effortful decision-making (Worbe *et al.*, 2015). This suggests that decreased serotonergic neurotransmission should facilitate reflexive-optimal learning at the expense of reflective-optimal learning, which would be consistent with results from the current study.

Other studies have used acute administration of serotonin reuptake inhibitors to examine the effects of serotonin on learning and could be useful in this area of research as well (for a review, see Rogers, 2011). Additionally, participant genotype was not examined, and it is unknown if genotype status, such as 5-HTTLPR genotype, interacts with acetaminophen exposure to influence category learning. Studies investigating how acetaminophen interacts with genetic variants are needed to further elucidate the specific pathways through which acetaminophen influences these processes.

To our knowledge, this study is the first to examine the influence of acetaminophen on dual-learning systems. We found that reflective-optimal decision-making can be enhanced by acetaminophen. Convergence with other evidence suggests that this influence involves effects on serotonin neurotransmission. It is important to note that some of the analyses yielded non-significant results (e.g. overall accuracy for the reflective-optimal task did not differ between groups) and that other findings (e.g. acetaminophen reduces likelihood that criterion was met for the reflexive-optimal task) emerged from exploratory analyses. Thus, these findings should be considered preliminary and need to be interpreted with caution until they are replicated (Munafò *et al*., 2017). Nevertheless, these results suggest that acetaminophen encourages effortful, deliberative learning at the expense of associative, implicit forms of information processing. Implications of this pattern are worth exploring further.

## Funding

This study was supported by funding from the National Institute on Drug Abuse (R01DA032457) to C.G.B.

*Conflict of interest.* The content is solely the responsibility of the authors and does not necessarily represent the official views of the National Institutes of Health.

## Footnotes

1 Consistent with prior work, we initially analyzed differences in trial level accuracy in a mixed model regression with fixed effects for trial number, experimental group and task condition, and a random intercept for participant. This interaction did reach statistical significance (b = 3.25, SE = 0.68, z = 4.76, *P* < 0.0001); however, for the reasons described, we believe the most appropriate model allows for a random slope and fixed intercept.
